# Understanding Participation in Genetic Research Among Patients With Multiple Sclerosis: The Influences of Ethnicity, Gender, Education, and Age

**DOI:** 10.3389/fgene.2020.00120

**Published:** 2020-03-13

**Authors:** Michael L. Cuccaro, Clara P. Manrique, Maria A. Quintero, Ricardo Martinez, Jacob L. McCauley

**Affiliations:** ^1^ John P. Hussman Institute for Human Genomics, University of Miami Miller School of Medicine, Miami, FL, United States; ^2^ Dr. John T. Macdonald Foundation, Department of Human Genetics and Genomics, University of Miami Miller School of Medicine, Miami, FL, United States

**Keywords:** participation, genetics, research, minorities, motivation, multiple sclerosis

## Abstract

This study examined reasons for participation in a genetic study of risk for multiple sclerosis (MS). Our sample consisted of 101 patients diagnosed with MS who were approached about enrolling in the Multiple Sclerosis Genetic Susceptibility Study. Participants were predominantly Hispanic (80%), female (80%), and well educated (71%), having at least some level of college education. Of these 101 individuals who were approached, 95 agreed to participate and are the focus of this report. Among enrollees, the most frequently cited reasons for participation were to find a cure for MS (56%), having MS (46%), and helping future generations (37%). Regression models comparing ethnic groups, Hispanics endorsed having MS as a reason to participate significantly more frequently than non-Hispanics (HI 52%, non-HI 19%, p = 0.015) while non-Hispanics endorsed finding new and better treatments significantly more frequently than Hispanics (Hispanic 17%, non-Hispanic 50%, p = 0.003). Among our three age groups, younger individuals endorsed finding a cure for MS significantly more frequently (74% of 18–35-year olds vs. 56% of 36–55 year olds vs. 39% of >55 year olds). Our results suggest that motivations for participation in genetic research vary by ethnicity, and that these influences need to be considered in developing more inclusive programs of disease-related genetic research. Future efforts should focus on development of standard methods for understanding participation in genetic and genomic research, especially among underrepresented groups as a catalyst for engaging all populations.

## Introduction

It is widely believed that underrepresented groups are less willing to participate in biomedical research due to barriers such as mistrust, stigma, and competing demands, leading to under-representation (Shavers et al., 2002; George et al., 2014). However, under-representation in biomedical research is also a by-product of limited access to research opportunities and reduced invitations to participate (Wendler et al., 2006; Katz et al., 2007), which persists to this day (Jones et al., 2017). Thus, even in situations where willingness to participate in biomedical research among underrepresented populations is indistinguishable from other groups, levels of participation may differ for other reasons (Katz et al., 2009; Fisher and Kalbaugh, 2011). Importantly, it is not clear that underrepresented groups’ attitudes about participation in biomedical research extend to participation in genetic research. Reduced willingness to participate in genetic research has generally been attributed to unfavorable attitudes about this type of research (Matsui et al., 2005). Clearly, there is much to be learned about why individuals from underrepresented populations participate in genetic research.

Among underrepresented populations, consistent themes for participation include altruism, benefit to family members, self-benefit, and personal curiosity (Sanderson et al., 2013; Walker et al., 2014). Similarly, concerns about individual and family health as well as helping the common good were primary motivations for participation in genetic research among African Americans enrolled in the Jackson Heart study (Walker et al., 2014). Respondents in this study also reported being motivated by the opportunity to get involved in something that would help African Americans across the country; most expressed a high confidence and trust in the study leaders and staff. Sanderson and colleagues conducted structured interviews to assess willingness to participate in genomics research on complex diseases among a diverse group of participants from an inner-city hospital, which included black, Hispanic, and non-Hispanic white individuals (Sanderson et al., 2013). Results showed that willingness to participate was motivated by altruism, benefit to family members, personal health benefit, personal curiosity and improving understanding. In contrast, unwillingness to participate was motivated by negative perceptions of research, lack of perceived personal relevance, negative feelings about procedures (e.g., blood draws), practical barriers, and fear of results (Sanderson et al., 2013).

The importance of participation in genetic research has implications for translational benefits associated with such research. For various groups that may already be under-served, an under-representation in genetic research can amplify future health disparities. For instance, Bustamante and colleagues report that failure to investigate a “broader ensemble of populations” will bias findings from genomic research and benefit only the privileged segment of the population who participate (Bustamante et al., 2011). While this situation has improved somewhat, there is still an underrepresentation of non-European populations in genetic research, which is crucial to ensuring that the benefits of research are available for all (Popejoy and Fullerton, 2016). The importance of genetics for health services has been anticipated for some time (Sterling et al., 2006). More than 10 years after Sterling and colleagues described the importance of genetics for health services (Sterling et al., 2006), the integration of genetics in health services has arrived as whole exome and whole genome sequencing technologies are increasingly present in clinical settings (Biesecker and Green, 2014; Krier et al., 2016). However, as noted by Landry and colleagues, a lack of equitable representation in this new era of precision medicine research will inhibit translational benefits for groups not represented (Landry et al., 2018).

Efforts to include underrepresented groups in genetic and genomic research have increased, albeit slowly. One line of study has examined influences on willingness to participate, including motivations. To date, findings from studies of motivation to participate in genomic research among underrepresented populations have been mixed, and some of the observed differences in outcomes may be attributable to study design. For example, some studies assess motivations to participate among individuals who enroll or decline participation in a genetic risk study (i.e., actual participation) (Parikh et al., 2017) while others survey intentions to participate (Halbert et al., 2016; Cooke Bailey et al., 2018). Similarly, some studies enroll patients who are from the general population of patients in both hospital and non-hospital setting (Sanderson et al., 2013; Walker et al., 2014; Jones et al., 2017), while others assess factors associated with participation among patients with specific diseases (Parikh et al., 2017). This is an important distinction as motivational factors vary considerably depending on the type of study and population (e.g., clinical trial vs. observational study, disease group vs. healthy population) (Goodman et al., 2018; Goodman et al., 2019). Further, the set of reasons that motivate healthy individuals to participate is likely very different from reasons that motivate individuals with specific diseases. To date, there have been limited studies using methods which directly ask individuals with specific diseases about reasons for participating in genetic research for those diseases. Acknowledging the concerns raised by Goodman and colleagues around conflating disease and healthy population studies and methods, we believe that asking patients who enroll in genetic studies about their reasons for enrollment is the most informative approach. This belief is supported by the work of the Clinical Sequencing Exploratory Research (CSER) consortium, which has investigated multiple facets of participation in genomic research, including why patients decline to participate (Amendola et al., 2018).

For this study, we asked patients with multiple sclerosis (MS) who were participating in a genetic risk study for MS to identify the primary reasons or motivations for participation using questions based on information from prior qualitative studies. We examined the frequencies of responses in relation to ethnicity, age, and gender. To date, incorporating genetics into precision medicine for MS is a work in progress (Giovannoni, 2017; Hansen and Okuda, 2018), but there has been considerable progress over the past several years (Matthews, 2015). As these genetic discoveries slowly accrue and become clinically useful, it is equally important that they are applicable across populations (Hindorff et al., 2018; Bonham et al., 2018). However, as noted above, the utility of genomic information in clinical settings rests on a foundation of established findings from prior studies and the absence of such information affects interpretation of clinical findings. Thus, a lack of diversity in research has the potential to exacerbate existing inequalities in health care (Popejoy and Fullerton, 2016). Given the under inclusion of non-European ancestry groups in genetic and genomic research, a necessary first step is to understand the factors that influence participation and then use this information to create more inclusive ascertainment.

## Methods

### Human Subjects Research Compliance

All procedures followed were in accordance with the ethical standards of the Institutional Review Board at the University of Miami Miller School of Medicine, and with the Helsinki Declaration of 1975, as revised in 1999 (Human, 1999). Informed consent was obtained from all participants included in the study.

### Participants and Enrollment

Participants for this study consisted of 101 patients with a diagnosis MS who were ascertained through the University of Miami Health System’s MS Center of Excellence, as well as the local community. Patients were eligible for this study if they had a clinical diagnosis of MS and were 18 years of age or older.

Potential enrollees in the genetic risk for MS study were recruited in the clinic setting or at a community outreach events, at which time they were invited to participate. Most of our participants were enrolled in the clinic setting, indicative of the volume of patients available at that site. Once they indicated their decision, the clinical coordinator would ask individuals to select a reason(s) for their decision (i.e., to participate in the genetic research study or not) from a list of possible reasons (which were presented to the participant) and record their answers. Participants also provided socio-demographic information at that time. All materials were presented in the preferred language of the participant.

### Measures

#### Sociodemographic information

Participants were asked their gender, race-ethnicity, and religious affiliation. In addition, they were asked to indicate their age group and education level.

#### Reasons for participation

We identified 11 possible reasons for participation (two of which were “other” and “not sure”) in a genetic research study (see list of reasons in **Supplementary Information**). The reasons were derived from multiple studies of reasons for participating in biomedical research (e.g., clinical trials and observational studies) as well as biobank and genetic studies (Streicher et al., 2011; Lang et al., 2013; Sanderson et al., 2013; Walker et al., 2014) that were primarily conducted among convenience samples of individuals with no known disease or illness. Given the paucity of published methods for evaluating willingness to participate in clinical populations we created questions that reflected the primary themes from other types of qualitative research (e.g., structured interviews and focus groups) that assessed willingness to participate in genetic research for reasons such as altruism (e.g.,*To help future generations*), personal benefit (e.g., *I suffer from MS*), and advancing research (e.g., *To help improve science and knowledge about MS*). The questions were drafted by one of the investigators (clinical psychologist) and subsequently reviewed by other team members including the director of patient and family ascertainment and senior clinical coordinators, both who have extensive experience in participant recruitment. Following revisions, the survey was administered to various staff to evaluate wording, item order, and item complexity.

### Data Analysis

Our primary questions of interest involved whether endorsement of reasons for participating in the genetic risk for MS study differed by ethnicity, gender, education, and age. To answer these questions, we conducted separate logistic regression analyses using ethnicity, gender, and education as binary outcomes (i.e., Hispanic vs. non-Hispanic, male vs. female, any college vs. no college), and our survey items as predictor variables. For age, we conducted multinomial logistic regression with three levels of our outcome variable (young = 18-35 years, middle = 36-55 years, and older = > 55 years). We tested each of the models for significance and report on those items which are significant contributors to the respective models (i.e., which items predict the outcomes of interests (e.g., Hispanics vs non-Hispanics), thereby reducing the number of significance tests to those associated with the four overall tests (corrected significance level *p* = 0.0125). Odds ratios and confidence intervals are available for each model. All statistical analyses were performed using SPSS version 24 software (SPSS, 2013) and were restricted to individuals who agreed to participate (n = 95).

## Results

Among the 101 individuals approached about participating in the genetic risk for MS study, 95 (94%) agreed to participate. All results are based on this group of 95 individuals. As seen in **Table 1**, most of our participants were Hispanic (N = 79; 83%) and female (N = 78; 82%). We tested whether our Hispanic and non-Hispanic participants differed with respect to gender and found no differences in the proportions of males and females by ethnicity (Fisher’s Exact Test, *p* = 0.15). Similarly, while a large percentage of the sample was college educated (71%), we found that our Hispanic and non-Hispanic participants did not differ in education (*p* = 0.58). Finally, there were no differences in age by ethnic group (*p* = 0.47)

**Table 1:** Cohort description (N=95).

Ethnicity	
Hispanic	N=79 (83%)
Non-Hispanic	N=16 (17%)
Sex	
Female	N=78 (82%)
Male	N=17 (18%)
Age	
18–35 years	N=23 (24%)
36–55 years	N=45 (48%)
> 55 years	N=26 (28%)
Education	
College	N=66 (70%)
Non-College	N=29 (30%)
Recruitment Site	
Clinic	N=78 (82%)
Home	N=12 (13%)
Other	N=5 (5%)

**Figure 1:**
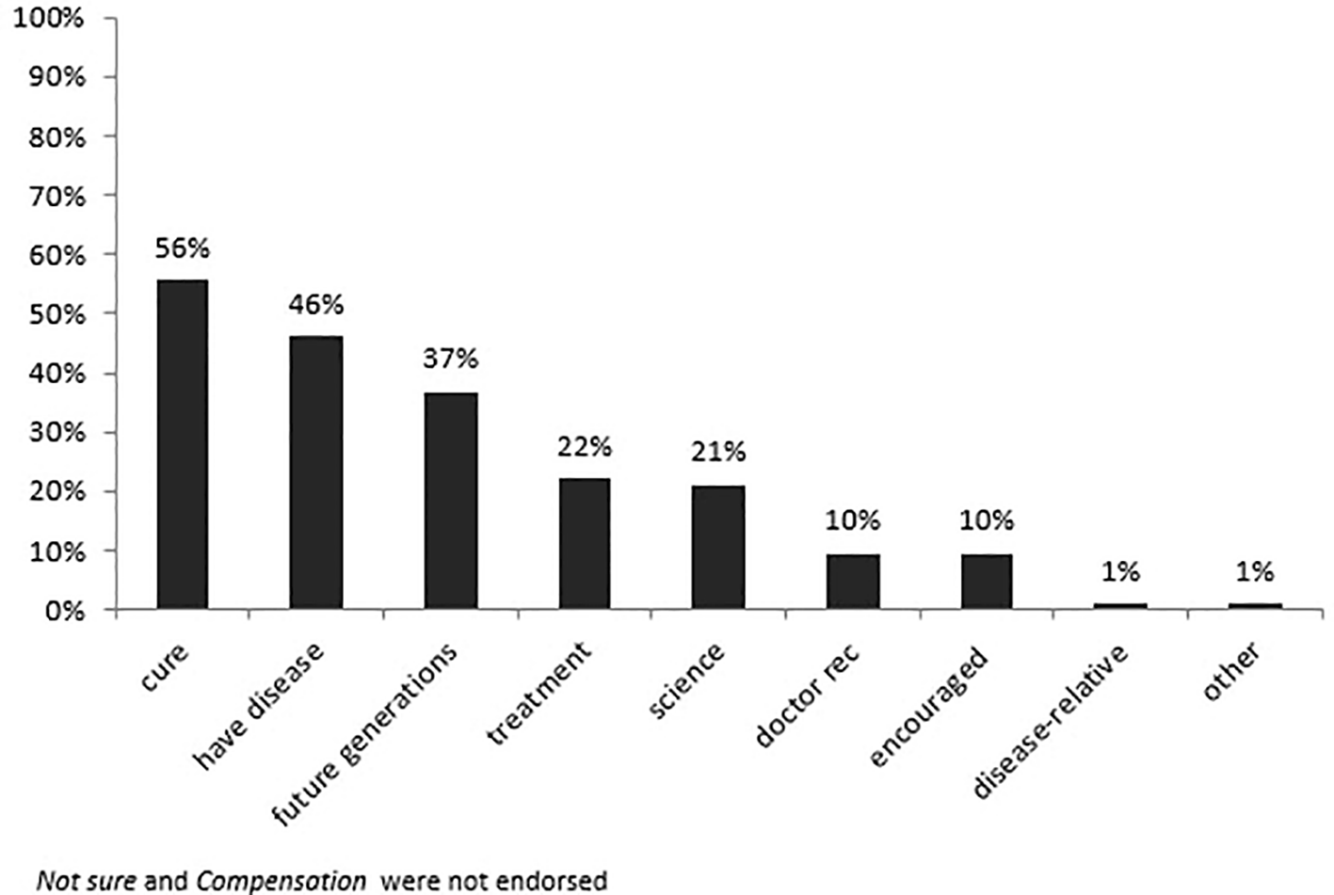
Percentage of endorsements per the respective reasons for participation in the overall sample.

Examination of overall endorsement patterns (**Figure 1**) showed that finding a cure, endorsed by 56% of participants, was the most frequently cited reason for participating in the study. In addition, having MS and helping future generations, were endorsed by a majority of participants as reasons to enroll in the MS study.

**Table 2** summarizes the endorsement patterns for the respective items by ethnicity, gender, education, and age. At the descriptive level, inspection of the frequencies of endorsements shows that both Hispanic and non-Hispanic participants cited finding a cure equally (56% per group). This was the most common reason for the respective groups. However, compared to non-Hispanics, Hispanic participants endorsed having a disease as a reason to participate in the genetic risk for MS study more frequently than non-Hispanics (HI 52%, NH 19%). Conversely, non-Hispanic participants cited finding new/better treatments more frequently than Hispanics (NHI 50%, HI 17%).

**Table 2:** Percentage of endorsements for reasons to participate by ethnicity, sex, education, and age (N and % values).

	Ethnicity		Sex		Education		Age (years)		
	HI N=79	NH N=16	M N=17	F N=78	College N=66	~College N=29	young N=23	middle N=45	old N=26
Cure for MS	44 56%	9 56%	6 35%	47 60%	39 59%	14 48%	17 74%	25 56%	10 39%
Suffer from MS	41 52%	3 19%	7 41%	37 47%	32 49%	12 41%	10 44%	22 49%	11 42%
Help future generations	27 34%	8 50%	5 29%	30 39%	25 38%	10 34%	7 30%	17 38%	10 39%
Better treatments for MS	13 17%	8 50%	5 29%	16 21%	17 26%	4 14%	7 30%	10 22%	3 12%
Improve science	15 19%	5 31%	3 18%	17 22%	15 23%	5 17%	6 26%	10 22%	3 12%
Recommended by Doctor	9 11%	0 –	2 12%	7 9%	6 9%	3 10%	1 4%	4 9%	4 15%
Encouraged by others	6 8%*	3 19%*	3 18%	6 8%	3 5%	6 21%	3 13%	3 7%	3 12%

Young=18–35 years.

Middle=36–55 years.

Old= > 55 years.

Endorsement patterns by sex, age, and education were similar to those identified in our ethnic groups as finding a cure and having multiple sclerosis were endorsed consistently as reasons for participating in the MS study.

To test for differences in reasons for participating in genetic research we conducted separate logistic regressions to ascertain the effects of the respective survey items (i.e., reasons for participating) on different binary (ethnicity, sex, and education groups) and multinomial (age groups) outcomes. For each of the respective analyses, we restricted our predictors to the following survey items: *I want to help find a cure for MS*; *To help improve science and knowledge about MS*; *To find new/better treatments for MS*; *I suffer from MS*; *To help future generations*; *The doctor asked/recommended that I participate*; *and, Encouragement from a family member or friend*. The remaining items were not cited as reasons for participating by more than one individual.

### Ethnic Group

Our logistic regression model evaluating the ability of survey items to predict ethnic group (Hispanic vs. non-Hispanic) was statistically significant, *χ^2^*(6) = 20.61, *p* = 0.002. Of the six predictor variables (i.e., survey items that were reasons for participating in the study), three contributed significantly to the model: *I suffer from MS, To find new/better treatments for MS*, and *Encouragement from a family member or friend*. These items differed between our Hispanic and non-Hispanic participants. Among the three items, the largest OR (7.34; CI 1.52, 35.68) was found for the item, *I suffer from MS*, indicating that endorsing this item as a reason was more likely among Hispanics vs. non-Hispanics. Conversely, *To find new/better treatments for MS* (OR = 0.15), and *Encouragement from a family member or friend* (OR = 0.13), were associated with a reduced likelihood of endorsement by Hispanics vs. non-Hispanics. **Table 3** has the odds ratios and confidence intervals for these results.

**Table 3:** Summary of logistic regression model for ethnic group (Hispanic vs non-Hispanic) using reasons for participation as predictors (predicted outcome=Hispanic).

	*B*	S.E.	Wald	*df*	*p*	OR	95% CI for OR	
							Lower	Upper
Cure for MS	.195	.681	.082	1	0.775	1.215	.320	4.619
Improve science	-.709	.844	.704	1	0.401	.492	.094	2.576
Better Treatments for MS*	-1.871	.723	6.708	1	0.010	.154	.037	.634
Suffer from MS*	1.995	.806	6.135	1	0.013	7.356	1.517	35.677
Help future generations	-1.021	.692	2.179	1	0.140	.360	.093	1.397
Encouraged by others*	-2.058	.959	4.607	1	0.032	.128	.019	.836

OR, odds ratio *significant coefficients.

MS, multiple sclerosis.

### Sex

The logistic regression model evaluating the ability of survey items to predict sex was not significant, *χ^2^*(7) = 6.54, *p* = 0.478, as none of the items differed between males and females. The odds ratios and confidence intervals for the respective items are available in Supplementary material (**Supplementary Table 1**).

### Education

Similar to the logistic regression model for sex, the model which evaluated the ability of survey items to predict educational group (college vs. no college) was not significant, *χ^2^*(7) = 7.33, *p* = 0.396). The odds ratios and confidence intervals for the respective items are also available in Supplementary material (**Supplementary Table 2**).

### Age

As seen in **Table 2**, we collapsed the various age groups into three categories (18–35 years of age, 36–55 years of age, and >55 years of age). Assessment of how well the model fits using likelihood ratio tests was not significant *χ^2^*(14) = 13.23, *p* = 0.508. For one of the predictors, we observed a trend in comparison of the older and younger groups (*p* = 0.021) although given that the omnibus test was not significant, this finding did not survive correction for multiple tests. However, the odds for selecting this as a reason to participate among younger vs. older participants was 4.896, 95% CI 1.28, 18.79) suggesting that this item is more likely among younger vs. older participants. These results along with the additional parameter estimates are available in supplementary material (**Supplementary Table 3**).

## Discussion

Overall, our logistic regression analyses yielded only one significant model which showed that there were different reasons for participating in genetic research between Hispanics and non-Hispanics. Among the reasons for participating, personal experience with MS (i.e., *I suffer from MS*), was strongly associated with Hispanics vs. non-Hispanics with an odds ratio of 7.36. In contrast, non-Hispanics were significantly more likely to endorse helping to discover new treatments (OR = 0.15) as a reason to participate. While personal experience with MS and discovery of new treatments are generally aligned with a theme of deriving personal benefit, the differences may hint at subtle distinctions between Hispanics and non-Hispanics or how the items were interpreted. Certainly, our findings regarding Hispanics being motivated by having a disease (i.e., MS) are in line with prior research showing that Hispanics are more likely to participate in biomedical research if it is relevant to them (Ulrich et al., 2013). Note that one additional item, encouragement from others (OR = 0.13), was less likely to be endorsed by Hispanics as a reason to participate in genetic research—again possibly reflecting personal motivation. The second item, finding new and better treatments, was endorsed by 50% of non-Hispanics vs. only 17% of Hispanics, and has elements of personal benefit as well as altruism. Further, while not significant, 50% of non-Hispanics endorsed helping future generations as a reason for motivation compared to 34% of Hispanics. Even though this difference was not significant, when coupled with the results regarding the item finding new and better treatments, there is a suggestion that Hispanics and non-Hispanics with MS may have different perspectives on what they see as priorities for participation.

Importantly, while interpretations of the above response patterns are reasonable and fit with previously published findings regarding personal meaningfulness and benefit to society (Goodman et al., 2019), we would encourage caution in interpretation of the results. In particular, given that we only asked participants to indicate if a particular reason motivated them to participate, endorsements could be interpreted in multiple ways. For instance, endorsement of *I suffer from MS* as a reason to participate could simply be acknowledging that their participation is important for research vs. a desire to derive personal benefit. Ultimately, in the absence of open-ended responses that could explain participant reasoning, multiple inferences about the meaningfulness of the data are possible.

Interestingly, while not significant, 50% of non-Hispanics endorsed helping future generations as a reason for motivation compared to 34% of Hispanics. Even though this difference was not significant, when coupled with the results regarding the item finding new and better treatments, there is a suggestion that Hispanics and non-Hispanics with MS differ in altruism. One additional item, encouragement from others (OR = 0.13), was less likely to be endorsed by Hispanics as a reason to participate in genetic research—again reflecting personal motivation.

At a descriptive level, our results show that among enrollees in an MS genetic risk study, the most frequently cited reason for participating was finding a cure for MS. While this reason for participation did not differ by ethnicity, sex, or education there was a trend among participants in different age groups. Specifically, for the item, *I want to help find a cure for MS*, a positive response was more likely among younger (i.e., 18-35 year olds) vs older (> 55 years) participants; our middle age group (36-55 years) did not differ from younger or older participants for this item. While it is not surprising that endorsement of finding a cure is high among respondents as a whole, especially given that seeking personal benefit is a powerful motivator for participation in biomedical and genetic research, an age-related effect has not been previously reported. Thus, while many studies adjust for age in their analyses to control for its influence on outcomes, this variable may be of value in terms of understanding the likelihood of participation. For instance, participants in the younger age groups may be more enthusiastic about finding a cure as they are still early in the disease process. At a minimum, investigators seeking to enroll participants for genetic studies should be aware of how age may affect motivations to participate in research when developing recruitment strategies.

The current study offers new information about motivations for participation in MS genetic research as a function of ethnicity and age. While the strengths of the study are its focus on individuals who have a disorder (MS) vs a hypothetical scenario, and the inclusion of Hispanics, the results should be interpreted with caution in light of several factors including small sample size, higher education levels, and a high rate of willingness to participate, raising the possibility of bias related to their being approached during a clinical encounter (i.e., at a neurology appointment). Consequently, our results may not be generalizable to individuals with MS who are receiving services outside of academic medical centers or those who are not receiving care. Moving forward, collecting more information such as duration and severity of illness, acculturation, and trust in the health care system, could reveal subtle influences on reasons for participation in genetic research. Finally, as noted in the *Methods* section, we developed the items (i.e., reasons for participation) based on themes from qualitative research conducted with mainly non-disease populations. Given the preliminary nature of our study, the questions have limited formal validation data. However, given the interesting results, we are expanding our efforts to learn more about participant motivations by providing participants an opportunity to explain their choices and recruiting both healthy individuals and those with diseases to compare response patterns. We believe these efforts will increase our ability to understand the nuances of why individuals participate in genetic studies and if those reasons vary by race and ethnicity.

In summary, this study adds to our understanding of influences on actual participation in research studies about genetic risk. Based on our study, it appears that ethnicity was the only significant factor associated with willingness to participate. Studies like this and others provide valuable information about why individuals ultimately participate in genetic research and can inform the development of recruitment strategies. Inclusive enrollment is critical to translational efforts that can play a major role in improving the health and well-being of all individuals.

## Data Availability Statement

The datasets generated for this study are available on request to the corresponding author.

## Ethics Statement

This research was approved by the Institutional Review Board, University of Miami Miller School of Medicine. MC, CM, MQ, RM, and JM declare that they have no conflict of interest. All procedures followed were in accordance with the ethical standards of the responsible committee on human experimentation (institutional and national) and with the Helsinki Declaration of 1975, as revised in 2000 (5). Informed consent was obtained from all patients included in the study.

## Author Contributions

MC, CM, MQ, RM, and JM contributed to the design and implementation of the research, to the analysis of the results, and to the writing of the manuscript.

## Funding

The research reported in this publication was supported by the National Institutes of Health (NIH) through the National Institute of Neurological Disorders and Stroke (NINDS) under award number 1R01NS096212, the National Institute on Minority Health and Health Disparities (NIMHD) and the National Human Genome Research Institute (NHGRI) under award number U54MD010722, and the National Multiple Sclerosis Society (NMSS) under award number RG4680A1. All content is solely the responsibility of the authors and does not necessarily represent the official views of the NIH or the NMSS.

## Conflict of Interest

The authors declare that the research was conducted in the absence of any commercial or financial relationships that could be construed as a potential conflict of interest.

The reviewer MM and handling Editor declared their shared affiliation.
