# Supplementary material for: Understanding Participation in Genetic Research Among Patients With Multiple Sclerosis: The Influences of Ethnicity, Gender, Education, and Age
Source: Front Genet. 2020 Mar 13;11:120. doi: 10.3389/fgene.2020.00120 (PMC7082924; doi:10.3389/fgene.2020.00120)
Supplement: Supplementary file 1 [file DataSheet_1.docx]

**Supplementary Information**

Reasons for participation

1. I want to help find a cure for MS

2. To help improve science and knowledge about MS.

3. To find new/better treatments for MS.

4. I suffer from MS.

5. I have a relative who suffers from MS.

6. To help future generations.

7. The doctor asked/recommended that I participate.

8. I am being paid for participating.

9. Encouragement from a family member or friend.

10. Not sure

11. Other
