# Supplementary material for: Understanding Participation in Genetic Research Among Patients With Multiple Sclerosis: The Influences of Ethnicity, Gender, Education, and Age
Source: Front Genet. 2020 Mar 13;11:120. doi: 10.3389/fgene.2020.00120 (PMC7082924; doi:10.3389/fgene.2020.00120)
Supplement: Supplementary file 2 [file Table_1.docx]

| **Supplementary-Table 1**. Summary of logistic regression model with sex as the outcome using reasons for participation as predictors (predicted outcome=male). | | | | | | | | |
| --- | --- | --- | --- | --- | --- | --- | --- | --- |
|  | *B* | S.E. | Wald | *df* | *p* | OR | 95% CI for OR | |
|  |  |  |  |  |  |  | Lower | Upper |
| Cure for MS | -1.075 | .603 | 3.184 | 1 | 0.074 | .341 | .105 | 1.112 |
| Improve science | .081 | .765 | .011 | 1 | 0.916 | 1.084 | .242 | 4.852 |
| Better treatments for MS | .747 | .666 | 1.257 | 1 | 0.262 | 2.110 | .572 | 7.783 |
| Suffer from MS | -.290 | .587 | .243 | 1 | 0.622 | .749 | .237 | 2.365 |
| Help future generations | -.444 | .625 | .505 | 1 | 0.477 | .641 | .188 | 2.184 |
| Encouraged by others | .083 | .927 | .008 | 1 | 0.928 | 1.087 | .177 | 6.688 |
| Recommended by doctor | .806 | .833 | .938 | 1 | 0.333 | 2.240 | .438 | 11.451 |
| OR=odds ratio  MS=multiple sclerosis | | | | | | | | |
