# Supplementary material for: Understanding Participation in Genetic Research Among Patients With Multiple Sclerosis: The Influences of Ethnicity, Gender, Education, and Age
Source: Front Genet. 2020 Mar 13;11:120. doi: 10.3389/fgene.2020.00120 (PMC7082924; doi:10.3389/fgene.2020.00120)
Supplement: Supplementary file 3 [file Table_2.docx]

| **Supplementary-Table 2**. Summary of logistic regression model with educational group (college vs. no college) as the outcome using reasons for participation as predictors (predicted outcome=college). | | | | | | | | |
| --- | --- | --- | --- | --- | --- | --- | --- | --- |
|  | *B* | S.E. | Wald | *df* | *p* | OR | 95% CI for OR | |
|  |  |  |  |  |  |  | Lower | Upper |
| Cure for MS | .347 | .493 | .495 | 1 | 0.482 | 1.414 | .538 | 3.714 |
| Improve science | -.155 | .633 | .060 | 1 | 0.806 | .856 | .248 | 2.961 |
| Better treatments for MS | .626 | .646 | .940 | 1 | 0.332 | 1.871 | .527 | 6.635 |
| Suffer from MS | .336 | .493 | .464 | 1 | 0.496 | 1.399 | .532 | 3.676 |
| Help future generations | -.117 | .504 | .054 | 1 | 0.816 | .890 | .332 | 2.387 |
| Encouraged by others | -.089 | .806 | .012 | 1 | 0.912 | .915 | .189 | 4.437 |
| Recommended by doctor | -1.512 | .787 | 3.694 | 1 | 0.055 | .220 | .047 | 1.030 |
| MS=multiple sclerosis  OR=odds ratio | | | | | | | | |
