# Supplementary material for: Understanding Participation in Genetic Research Among Patients With Multiple Sclerosis: The Influences of Ethnicity, Gender, Education, and Age
Source: Front Genet. 2020 Mar 13;11:120. doi: 10.3389/fgene.2020.00120 (PMC7082924; doi:10.3389/fgene.2020.00120)
Supplement: Supplementary file 4 [file Table_3.docx]

| **Supplementary-Table 3**. Summary of multinomial logistic regression model with age group (young, middle, old) as the outcome using reasons for participation as predictors. | | | | | | | | |
| --- | --- | --- | --- | --- | --- | --- | --- | --- |
|  | | | | | | | 95% CI for OR | |
| Middle vs. Young | *B* | SE | Wald | *df* | *p* | OR | Lower Bound | Upper Bound |
| Cure for MS | .891 | .607 | 2.156 | 1 | 0.142 | 2.436 | .742 | 8.000 |
| Improve science | -.019 | .687 | .001 | 1 | 0.978 | .981 | .255 | 3.769 |
| Better treatments for MS | .608 | .647 | .885 | 1 | 0.347 | 1.837 | .517 | 6.524 |
| Suffer from MS | .093 | .565 | .027 | 1 | 0.870 | 1.097 | .362 | 3.324 |
| Help future generations | -.329 | .599 | .303 | 1 | 0.582 | .719 | .223 | 2.325 |
| Encouraged by others | 1.095 | .935 | 1.372 | 1 | 0.242 | 2.989 | .478 | 18.684 |
| Recommended by doctor | -.622 | 1.202 | .267 | 1 | 0.605 | .537 | .051 | 5.665 |
| Old vs. Young |  |  |  |  |  |  |  |  |
| Cure for MS | 1.588 | .686 | 5.360 | 1 | 0.021 | 4.896 | 1.276 | 18.786 |
| Improve science | .392 | .879 | .198 | 1 | 0.656 | 1.479 | .264 | 8.290 |
| Better treatments for MS | 1.389 | .850 | 2.671 | 1 | 0.102 | 4.013 | .758 | 21.237 |
| Suffer from MS | .568 | .662 | .737 | 1 | 0.391 | 1.765 | .482 | 6.455 |
| Help future generations | -.451 | .683 | .437 | 1 | 0.509 | .637 | .167 | 2.429 |
| Encouraged by others | .905 | .986 | .842 | 1 | 0.359 | 2.471 | .358 | 17.065 |
| Recommended by doctor | -1.110 | 1.244 | .795 | 1 | 0.372 | .330 | .029 | 3.777 |
| Old vs. Middle |  |  |  |  |  |  |  |  |
| Cure for MS | .698 | .542 | 1.655 | 1 | 0.198 | 2.009 | .694 | 5.819 |
| Improve science | .411 | .754 | .297 | 1 | 0.586 | 1.508 | .344 | 6.605 |
| Better treatments for MS | .781 | .749 | 1.089 | 1 | 0.297 | 2.184 | .504 | 9.472 |
| Suffer from MS | .475 | .539 | .779 | 1 | 0.378 | 1.608 | .560 | 4.622 |
| Help future generations | -.122 | .540 | .051 | 1 | 0.822 | .885 | .307 | 2.551 |
| Encouraged by others | -.190 | .906 | .044 | 1 | 0.834 | .827 | .140 | 4.881 |
| Recommended by doctor | -.488 | .806 | .367 | 1 | 0.545 | .614 | .127 | 2.978 |
| Young=18-35 years (reference) OR=odds ratio  Middle=36-55 years MS=multiple sclerosis  Old= >55 years | | | | | | | | |
